# *Escherichia coli* ST131: a multidrug-resistant clone primed for global domination

**DOI:** 10.12688/f1000research.10609.1

**Published:** 2017-02-28

**Authors:** Johann D.D. Pitout, Rebekah DeVinney

**Affiliations:** 1 Departments of Microbiology, Immunology, and Infectious Diseases, Cummings School of Medicine, University of Calgary, Calgary, Alberta, Canada; 2 Departments of Pathology and Laboratory Medicine, Cummings School of Medicine, University of Calgary, Calgary, Canada; 3 Division of Microbiology, Calgary Laboratory Services, Calgary, Alberta, Canada; 4 Department of Medical Microbiology, University of Pretoria, Pretoria, South Africa

**Keywords:** Escherichia coli ST131, ExPEC, antimicrobial-resistant infection

## Abstract

A single extra-intestinal pathogenic *Escherichia coli *(ExPEC) clone, named sequence type (ST) 131, is responsible for millions of global antimicrobial-resistant (AMR) infections annually. Population genetics indicate that ST131 consists of different clades (i.e. A, B, and C); however, clade C is the most dominant globally. A ST131 subclade, named C1-M27, is emerging in Japan and has been responsible for the recent increase in AMR ExPEC in that country. The sequential acquisition of several virulence and AMR genes associated with mobile genetic elements during the 1960s to 1980s primed clade C (and its subclades C1 and C2) for success in the 1990s to 2000s. IncF plasmids with F1:A2:B20 and F2:A1:B replicons have shaped the evolution of the C1 and C2 subclades. It is possible that ST131 is a host generalist with different accessory gene profiles. Compensatory mutations within the core genome of this clone have counterbalanced the fitness cost associated with IncF plasmids. ST131 clade C had dramatically changed the population structure of ExPEC, but it still remains unclear which features of this clade resulted in one of the most unprecedented AMR successes of the 2000s.

## Introduction

Extra-intestinal pathogenic *Escherichia coli*, or ExPEC, is a major human pathogen and is the most common cause of urinary tract infections and the most common Gram-negative bacterium associated with bloodstream infections in both developed and developing countries ^1^. Certain virulence factors (VFs) give ExPEC the means to cause disease. They include toxins, adhesions, lipopolysaccharides, capsules, proteases, and invasins and are important in colonization, which is often a precondition for virulence. However, the exact roles of these VFs have not been well defined.

Before the 2000s, ExPEC was mostly susceptible to first-line antibiotics (e.g. cephalosporins [cephs] and fluoroquinolones [FQs]) that are often used to treat infections ^2^. A recent World Health Organization report states that resistance to the FQs (FQ-R) among *E. coli* is very widespread and, in many parts of the world, FQs are now ineffective in more than half of patients ^3^. Of special concern is that FQ-R is often accompanied by resistance to the cephs (ceph-R), which is mainly due to the production of extended-spectrum β-lactamases (ESBLs), especially an enzyme named CTX-M-15 ^4^.

A single ExPEC clone, sequence type (ST) 131, is predominantly responsible for this global FQ-R and ceph-R pandemic causing millions of antimicrobial-resistant (AMR) infections annually (e.g. up to 30% of all ExPEC, 60–90% of FQ-R ExPEC, and 40–80% of ESBL ExPEC belongs to ST131) ^5^. Population genetics indicate that ST131 consists of different clades: clade A is associated mostly with *fimH*41, clade B with *fimH*22, and clade C with *fimH*30. The change in *fimH* alleles might improve colonization abilities of the different clades ^6^. Global longitudinal studies showed that clade B was presiding among ST131 before the 1990s, but since the 2000s clade C has become the most dominant lineage (currently up to 80% of global ST131 belongs to clade C) ^5^. Next-generation sequencing (NGS) identified two subclades within clade C named C1/H30R (associated with FQ-R) and C2/H30-Rx (associated with the ESBL CTX-M-15) ^6^. Both subclades showed extensive global distribution. The aim of this article is to update readers on recent evolutionary aspects regarding *E. coli* ST131.

## The sequential acquisition of several virulence and antimicrobial-resistance genes during the 1960 to 1980s primed ST131 for success in the 1990s to 2000s

The acquisition of certain key genomic islands (GIs) with VFs that pre-dated the development of FQ-R in clade C possibly played a role in the successful global dissemination of subclades C1 and C2. This probably required a previous enrichment in C subclades and might be secondary to the change in *fim* alleles. ST131 clade C is defined by high-level FQ-R mutations in *gyrA* ( *gyrA1AB*) and *parC* ( *parC1aAB*) and differs from clade B by 70 substitution single-nucleotide polymorphisms (SNPs) ^6^. Clade B is most often FQ susceptible and rarely carries plasmids with *bla*_CTX-M-15_, while clade C is mostly FQ-R and the C2 subclade is often associated with *bla*_CTX-M-15_^5^.

Recent phylogenetic studies from Oxford, UK, and Brisbane, Australia, have shed some light regarding the origin and evolution of ST131 clade C ^7, 8^. Both studies used NGS to characterize over 400 global ST131 from clinical, environmental, and veterinary sources. They showed that clade C evolved from clade B and this most likely occurred during the late 1980s in North America (either the United States or Canada) ^7, 8^.

**Figure 1.**
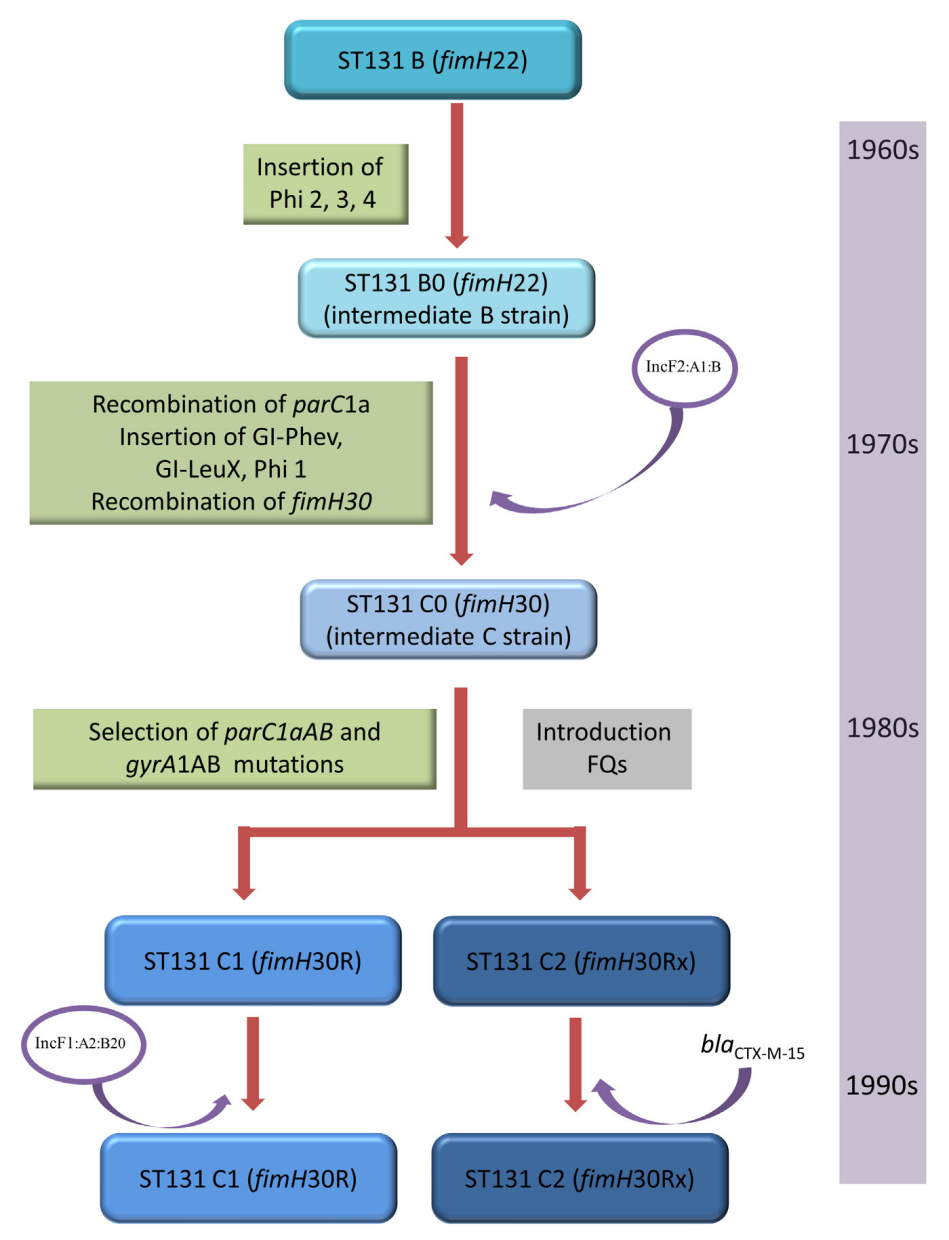
Stepwise evolution of *Escherichia coli* ST131 clades B, B0, C0, and C. FQ, fluoroquinolones; GI, genomic islands; Inc, incompatibility; Phi, prophages; ST, sequence types.

The Brisbane study also described a stepwise evolution process in which clade B was divided into five paraphyletic subclades and then sequentially acquired several prophages (Phi), GIs, the *fimH30* allele, and mutations within *gyrA* and *parC* and evolved into clade C ^8^. Each one of these acquisitions increased the population size and consequently extended the relational interactive field of the successive variants. They identified certain ST131 isolates that share characteristics of both clade B and clade C (these isolates are referred to as intermediate strains and named B0 [those isolates that shared more characteristics with clade B] and C0 [those isolates that shared more characteristics with clade C]). Analysis of clade B, intermediate strains B0 and C0, and subclades C1 and C2 identified intermediate patterns of recombination, primarily clustered around known mobile genetic elements, indicative of stepwise evolution among these intermediate strains (please refer to Figure 1 for details). The investigators from Brisbane noted the insertion of the Flag-2 locus in clade B, followed by the acquisition of different Phi (2, 3, and 4) leading to the emergence of intermediate strains B0. This process most likely occurred during the 1960s to 1970s. The authors then traced the acquisition of GI- *pheV* and GI- *leuX* and the recombination of *parC1a* to the most recent common ancestor of the intermediate strains C0 (i.e. *parC1a* is an allelic variant of the chromosomal *parC* gene that does not confer resistance to FQs). Strains that belonged to clade C0 were obtained several years before the emergence of subclades C1 and C2 (most likely during the late 1970s to early 1980s). GI- *pheV* is known to carry the autotransporter genes *agn-43* and *sat*, the ferric aerobactin biosynthesis gene cluster ( *iucABCD*), and its cognate ferric siderophore receptor gene *iutA*. The C clade-defining *fimH30* allele was then acquired by recombination, most likely during the early–mid 1980s, possibly in conjunction with the acquisition of the nearby GI *-leuX* ( Figure 1). The Oxford and Brisbane studies also showed that clade C instantly divided into subclades C1 and C2 after the acquisition of the high-level FQ-R mutations (via selection) in *parC (parC1aAB)* and *gyrA* ( *gyrA1AB*) that defined clade C ( Figure 1). This process transpired during the mid–late 1980s and coincided with the introduction of the FQs (especially ciprofloxacin) in clinical medicine.

These results suggest that the acquisition of virulence-associated genes (e.g. *sat* and *iutA*), AMR genes (i.e. *parC1a*), and *fimH30* in a stepwise process during the 1960s, 1970s, and early 1980s has primed ST131 for success prior to the acquisition of high-level FQ-R mutations in the late 1980s ( Figure 1). It is unclear if subclade C2 contained *bla*_CTX-M-15_ at that time (it did contain IncF plasmids that are associated with CTX-M-15 [details below]). A molecular epidemiology study from Calgary, investigating the prevalence of ST131 clades over time, showed that subclade C2 became prominent only towards the end of the 2000s, suggesting that it took some time for C2 to acquire *bla*_CTX-M-15_^9^.

## IncF plasmids have shaped the evolution of ST131 C1 and C2 clades

Epidemic resistance plasmids that belong to incompatibility (Inc) groups with F replicons (named IncF) have the ability to acquire resistance genes and rapidly disseminate among Enterobacteriaceae ^10^. IncF plasmids use post-segregation killing and addiction systems to ensure their propagation among high-risk clones such as ST131. The *bla*_CTX-M-15_ gene has mainly been found on certain IncF plasmids (especially with FIA-FII replicons) in ST131, whereas Inc plasmids with different replicons have been identified in non-ST131 ExPEC ^11, 12^. It has been postulated recently that the presence of IncF plasmids harboring *bla*_CTX-M-15_ is central to the global success of ST131 and that they have significantly contributed to the evolutionary dominance of subclade C2 ^13^. The study from Oxford has shown the clear relationship of subclade C2 with IncFII_AY458016 containing *bla*_CTX-M-15_ and the *pemI*/ *pemK* addiction system ^7^.

A study from Minnesota, USA, characterized plasmids in a collection of 104 diverse (i.e. clinical, environmental, and veterinary) ST131 isolates ^14^. They showed that the IncF variants differ among the C1 and C2 subclades: e.g. F1:A2:B20 replicons (without *bla*_CTX-M-15_) are associated with subclade C1, while F2:A1:B replicons (with *bla*_CTX-M-15_) are associated with subclade C2 ^19^ (the F2:A1:B replicons correlate with IncFII_AY458016 described in the Oxford study). They then proposed a sequence of events leading to the current circulating plasmids within the C subclades. The initial step was the introduction of an F2:A1 plasmid type (without *bla*_CTX-M-15_) into either clade B0 or clade C0 ( Figure 1). The C clade then evolved from C0 and separated into subclades C1 and C2 (as described above). Subclade C1 acquired the F1:A2:B2 plasmid, while the F2:A1:B plasmid in subclade C2 acquired AMR cassettes containing *bla*_CTX-M-15_, *catB4*, *bla*_OXA-1_, *aac(6')Ib-cr*, and *tetAR* via IS *26*-mediated events ( Figure 1). Subsequently, in some C2 isolates, *bla*_CTX-M-15_ was integrated into the bacterial chromosome, while in other isolates the AMR cassettes were lost over time. It seems that the C1 and C2 subclades have co-adapted with these plasmids to carry them at lower cost to the bacterial cell and that the plasmids themselves are evolving toward fixation within these clades, playing important roles in the success of their hosts. Toxin–antitoxin systems ensure the plasmids’ persistence in the clonal backgrounds in which they are located, preventing promiscuity among different clades/lineages.

## A different *E. coli* ST131 clade is emerging in Japan

ExPEC with *bla*_CTX-M-15_ is rare in Japan despite the predominance of ST131 among ESBL-producing isolates ^15^. Before 2005, ST131 clade C1 containing *bla*_CTX-M-14_ predominated among Japanese ST131 and since then has been replaced by clade C1 with *bla*_CTX-M-27_, which was responsible for a significant increase of ESBL-producing ExPEC in that country, especially since 2010 ^15^.

A study from Kyoto, Japan, performed NGS on 43 Japanese and 10 global ST131 isolates with *bla*_CTX-M-27_, *bla*_CTX-M-14_, and *bla*_CTX-M-15_ to investigate the emergence of ExPEC with *bla*_CTX-M-27_ in that country ^16^. The authors identified a discrete ST131:O75:H30 lineage that formed a distinct cluster within the C1 subclade and named it “C1-M27”, which was defined by a unique Phi-like region (M27PP1). Interestingly, subclade C1-M27 was responsible for the recent increase in ESBL-producing ExPEC from Japan and was also present among ST131 obtained from Thailand, Australia, Canada, and the USA, indicating that this subclade is not necessarily limited to Japan.

## *E. coli* ST131 is possibly a host generalist with different accessory gene profiles, and compensatory mutations have counterbalanced the fitness cost of plasmids

Previous clinical and ecological studies have shown that ST131 is relatively rare among animal ExPEC and seems to be an exclusively human pathogen ^5^. Several *in vitro* studies performed during the 2000s identified distinctive VF profiles among ST131, with the C2 subclade often having the highest aggregative virulence scores ^17^.

A multinational group of investigators analyzed the variations in the core, accessory, and regulatory genome regions in over 200 diverse ST131 isolates from avian species, domesticated animals, and humans to provide details on the ecology and evolution of this important ExPEC clone ^18^. ST131 strains isolated from wild birds, cats, and dogs were distributed throughout the phylogenetic tree and did not cluster separately to the human isolates. This suggested that ST131 has the ability to move easily between species and advocates that ST131 is a host generalist capable of frequent inter-species movement. However, current molecular epidemiological data do not support this finding; ST131 is rare among environmental and veterinary isolates.

Variations in the ST131 accessory gene pool identified by the multinational study showed the existence of multiple subtypes within subclades C1 and C2 based on highly similar accessory gene profiles. This might explain in part the different *in vitro* VF profiles of ST131 that had previously been described by various investigators ^17^.

AMR, either by mutation or the acquisition of resistance determinants on plasmids, confers a biological fitness cost to bacteria that can affect growth rate, survival abilities, and virulence capacities ^19^. Some bacteria can undergo compensatory mutations to reduce this fitness cost, which allows resistant isolates to adapt, flourish, and spread ^19^. The exact roles of compensatory mutations that offset the fitness costs associated with AMR are controversial and remain a relatively understudied area. Variations within gene regulatory regions of ST131 identified in the multinational study illustrated that the acquisition and maintenance of IncF plasmids by subclades C1 and C2 possibly occurred as a result of several compensatory mutations in the core genome of these isolates ^18^. These compensatory mutations influence gene expression and minimize the fitness costs associated with the maintenance of AMR IncF plasmids. This would, in part, explain the ability of ST131 to retain IncF plasmids over time, even in the absence of antibiotic selection pressure. It is important to remember that it is not experimentally proven and that IncF plasmids are, in general, common among *E. coli* populations and therefore “well evolutionarily adapted”.

## What makes *E. coli* ST131 so special?

The population structure of ST131 has been explored extensively by numerous research groups worldwide. In contrast, studies of the biological mechanisms that enabled the success of ST131 are largely lacking ^13^. It still remains unclear which features of ST131 clade C resulted in one of the most unprecedented AMR triumphs of the 2000s. The success of clade C was likely driven, in part, by the sequential acquisition of virulence factors, FQ-R, and ESBL production in an era when the use of FQs and oxyimino-cephs (especially the third-generation cephs) was increasing globally. The selection pressures created by the widespread use of these agents (i.e. FQs and oxyimino-cephs) have dramatically changed the population structure of ExPEC ^20^. However, there are other ExPEC strains and clones (e.g. ST405) with the same AMR determinants and similar virulence gene profiles as ST131 but that do not share the success of clade C ^5^.

Is *E. coli* ST131 clade C inherently more fit than other ExPEC clones or even other ST131 clades and therefore able to better survive in certain environments, even in the absence of antimicrobial selection pressures? Is this “fitness” due to certain VFs and compensatory mutations that provided clade C with opportunities to be exposed to and acquire certain IncF plasmids? It is also possible that the maintenance and co-evolution of subclades C1 and C2 with IncF plasmids containing F1:A2:B20 and F2:A1:B replicons, respectively, have provided rapid and continual adaptation opportunities for these subclades, providing them with the additional ability to outcompete other ExPEC clades. This is consistent with both the macro- and micro-evolutionary versions of the Red Queen hypothesis of co-evolution ^13^. It is important to remember that this might be a very simplistic view regarding the role of IncF plasmids in the success of ST13 and this clone frequently harbors non-F AMR plasmids.

This is reminiscent of a famous quote from Stephen Hawking: “intelligence is the ability to adapt to change”. ST131 clade C adapted to environmental changes more rapidly than other ExPEC clones. The medical community needs to know why and how. Without a better mechanistic understanding of the unique adaptations of this important clade, the medical community is unlikely to stop its continuing spread or to anticipate the next clonal wave of multidrug-resistant ExPEC.

## Abbreviations

AMR, antimicrobial resistance; cephs, cephalosporins; ceph-R, cephalosporin resistance; ESBL, extended-spectrum β-lactamase; ExPEC, extra-intestinal pathogenic *Escherichia coli*; FQs, fluoroquinolones; FQ-R, fluoroquinolone resistance; GI, genomic island; Inc, incompatibility; NGS, next generation sequencing; Phi, prophage; ST, sequence type; VF, virulence factor.

## References

[1] Pitout JD: Extraintestinal Pathogenic *Escherichia coli*: A Combination of Virulence with Antibiotic Resistance. *Front Microbiol.* 2012;3:9. 10.3389/fmicb.2012.00009 22294983 PMC3261549

[2] Pitout JD: Extraintestinal pathogenic *Escherichia coli*: an update on antimicrobial resistance, laboratory diagnosis and treatment. *Expert Rev Anti Infect Ther.* 2012;10(10):1165–76. 10.1586/eri.12.110 23199402

[3] World Health Organization: Antimicrobial resistance: global report on surveillance 2014. April 2014 ed, World Heath Organization,2014;257 Reference Source

[4] Pitout JD Laupland KB: Extended-spectrum beta-lactamase-producing Enterobacteriaceae: an emerging public-health concern. *Lancet Infect Dis.* 2008;8(3):159–66. 10.1016/S1473-3099(08)70041-0 18291338

[5] Nicolas-Chanoine MH Bertrand X Madec JY: *Escherichia coli* ST131, an intriguing clonal group. *Clin Microbiol Rev.* 2014;27(3):543–74. 10.1128/CMR.00125-13 24982321 PMC4135899

[6] Petty NK Ben Zakour NL Stanton-Cook M: Global dissemination of a multidrug resistant *Escherichia coli* clone. *Proc Natl Acad Sci U S A.* 2014;111(15):5694–9. 10.1073/pnas.1322678111 24706808 PMC3992628

[7] Stoesser N Sheppard AE Pankhurst L: Evolutionary History of the Global Emergence of the *Escherichia coli* Epidemic Clone ST131. *MBio.* 2016;7(2):e02162. 10.1128/mBio.02162-15 27006459 PMC4807372

[8] Ben Zakour NL Alsheikh-Hussain AS Ashcroft MM: Sequential Acquisition of Virulence and Fluoroquinolone Resistance Has Shaped the Evolution of *Escherichia coli* ST131. *MBio.* 2016;7(2):e00347–16. 10.1128/mBio.00347-16 27118589 PMC4850260

[9] Peirano G Pitout JD: Fluoroquinolone-resistant *Escherichia coli* sequence type 131 isolates causing bloodstream infections in a canadian region with a centralized laboratory system: rapid emergence of the *H*30-Rx sublineage. *Antimicrob Agents Chemother.* 2014;58(3):2699–703. 10.1128/AAC.00119-14 24566175 PMC3993220

[10] Carattoli A: Resistance plasmid families in *Enterobacteriaceae*. *Antimicrob Agents Chemother.* 2009;53(6):2227–38. 10.1128/AAC.01707-08 19307361 PMC2687249

[11] Shin J Choi M Ko KS: Replicon sequence typing of IncF plasmids and the genetic environments of *bla*CTX-M-15 indicate multiple acquisitions of *bla*CTX-M-15 in *Escherichia coli* and *Klebsiella pneumoniae* isolates from South Korea. *J Antimicrob Chemother.* 2012;67(8):1853–7. 10.1093/jac/dks143 22566590

[12] Zong Z: Complete sequence of pJIE186-2, a plasmid carrying multiple virulence factors from a sequence type 131 *Escherichia coli* O25 strain. *Antimicrob Agents Chemother.* 2013;57(1):597–600. 10.1128/AAC.01081-12 23070168 PMC3535984

[13] Mathers AJ Peirano G Pitout JD: The role of epidemic resistance plasmids and international high-risk clones in the spread of multidrug-resistant *Enterobacteriaceae*. *Clin Microbiol Rev.* 2015;28(3):565–91. 10.1128/CMR.00116-14 25926236 PMC4405625

[14] Johnson TJ Danzeisen JL Youmans B: Separate F-Type Plasmids Have Shaped the Evolution of the *H30* Subclone of *Escherichia coli* Sequence Type 131. *mSphere.* 2016;1(4): pii: e00121-16. 10.1128/mSphere.00121-16 27390780 PMC4933990

[15] Matsumura Y Johnson JR Yamamoto M: CTX-M-27- and CTX-M-14-producing, ciprofloxacin-resistant *Escherichia coli* of the *H30* subclonal group within ST131 drive a Japanese regional ESBL epidemic. *J Antimicrob Chemother.* 2015;70(6):1639–49. 10.1093/jac/dkv017 25687644

[16] Matsumura Y Pitout JD Gomi R: Global *Escherichia coli* Sequence Type 131 Clade with *bla*CTX-M-27 Gene. *Emerging Infect Dis.* 2016;22(11):1900–7. 10.3201/eid2211.160519 27767006 PMC5088012

[17] Banerjee R Robicsek A Kuskowski MA: Molecular epidemiology of *Escherichia coli* sequence type 131 and Its H30 and H30-Rx subclones among extended-spectrum-β-lactamase-positive and -negative *E. coli* clinical isolates from the Chicago Region, 2007 to 2010. *Antimicrob Agents Chemother.* 2013;57(12):6385–8. 10.1128/AAC.01604-13 24080662 PMC3837873

[18] McNally A Oren Y Kelly D: Combined Analysis of Variation in Core, Accessory and Regulatory Genome Regions Provides a Super-Resolution View into the Evolution of Bacterial Populations. *PLoS Genet.* 2016;12(9):e1006280. 10.1371/journal.pgen.1006280 27618184 PMC5019451

[19] Andersson DI Hughes D: Antibiotic resistance and its cost: is it possible to reverse resistance? *Nat Rev Microbiol.* 2010;8(4):260–71. 10.1038/nrmicro2319 20208551

[20] Banerjee R Johnson JR: A new clone sweeps clean: the enigmatic emergence of *Escherichia coli* sequence type 131. *Antimicrob Agents Chemother.* 2014;58(9):4997–5004. 10.1128/AAC.02824-14 24867985 PMC4135879

